# A minimally invasive method of piscine tissue collection and an analysis of long-term field-storage conditions for samples

**DOI:** 10.1186/1471-2156-7-32

**Published:** 2006-05-30

**Authors:** James J Campanella, John V Smalley

**Affiliations:** 1 Montclair State University, Department of Biology and Molecular Biology, 1 Normal Avenue, Upper Montclair, New Jersey 07043, USA; 2 Bergen Community College, Department of Science and Technology, 400 Paramus Road, Paramus, New Jersey 07652, USA

## Abstract

**Background:**

The acquisition of high-quality DNA for use in phylogenetic and molecular population genetic studies is a primary concern for evolutionary and genetic researchers. Many non-destructive DNA sampling methods have been developed and are used with a variety of taxa in applications ranging from genetic stock assessment to molecular forensics.

**Results:**

The authors have developed a field sampling method for obtaining high-quality DNA from sunfish (*Lepomis*) and other freshwater fish that employs a variation on the buccal swab method and results in the collection of DNA suitable for PCR amplification and polymorphism analysis. Additionally, since the circumstances of storage are always a concern for field biologists, the authors have tested the potential storage conditions of swabbed samples and whether those conditions affect DNA extraction and PCR amplification. It was found that samples stored at room temperature in the dark for over 200 days could still yield DNA suitable for PCR amplification and polymorphism detection.

**Conclusion:**

These findings suggest that valuable molecular genetic data may be obtained from tissues that have not been treated or stored under optimal field conditions. Furthermore, it is clear that the lack of adequately low temperatures during transport and long term storage should not be a barrier to anyone wishing to engage in field-based molecular genetic research.

## Background

The acquisition of high quality DNA for use in phylogeny and population genetic studies is a prime concern for researchers. While such DNA samples are easily obtained, it often requires the sacrifice of the animals being studied. Such lethal sampling has the potential to seriously impact the genetic makeup of populations under investigation, altering the composition of the future population.

A number of non-destructive nucleic acid sampling methods have been developed with a variety of taxa in applications ranging from genetic stock assessment to molecular forensics. DNA samples suitable for PCR amplification and analysis of polymorphisms in honey bees (*Apis mellifera*) have been obtained from wing clips [1]. Toe clips have been used to obtain samples of DNA for genetic studies of the Great Plains toad, *Bufo cognatus *[2]. In piscine species, tissue sources of DNA for non-lethal sampling include: fin clips, scales, barbels, muscle, blood, and sperm [3,4]. The molecular phylogeny of the family *Chinchillidae *has been investigated using DNA from hair, blood, feces, and ear tissue [5]. DNA suitable for microsatellite analysis and genotyping has even been obtained from Amur tiger (*Panthera tigris altaica*) and chimpanzee (*Pan troglodytes*) feces [6,7], and from long-dead sperm whales (*Physeter macrocephalus*) using teeth and scrimshaw [8].

A standard method of collecting DNA from humans involves the minimally invasive process of buccal swabbing to separate away epithelial cells from which the DNA is then extracted [9,10]. Among the advantages of this method are its swiftness and ease of application [11]. These characteristics make buccal swabbing adaptable to a wide variety of situations and particularly amenable to large sample sizes.

In addition to the problem of collecting tissue samples non-destructively, the storage of said samples in the field for subsequent DNA extraction presents a difficulty for the field biologist involved in molecular analysis. Since elaborate DNA extraction methods that would ensure sample stability usually can not be employed in the field, alternatives must be found. Low temperature tissue storage is an obvious, although potentially impractical, solution. Tissues fixed in 50% ethanol have yielded DNA suitable for restriction endonuclease digestion and hybridization with oligonucleotide probes after storage for 6 years [12]. Storage of tissues in a Lysis/Storage/Transportation (LST) buffer at room temperature for up to 8 weeks has allowed extraction of DNA of sufficient quality for restriction endonuclease digestion as well as PCR amplification [13].

The authors have developed a field sampling method for obtaining high quality DNA from bluegill sunfish (*Lepomis macrochirus*). This method employs a variation on the buccal swab method and results in the collection of DNA suitable for PCR amplification and polymorphic analysis. Additionally, we address the question of how robust our method would be for a field scientist who did not have the proper storage facilities. If a field scientist did not have proper refrigeration, would tissues sampled by buccal swabbing be stable and, if so, for how long?

## Results and discussion

**Figure 1:**
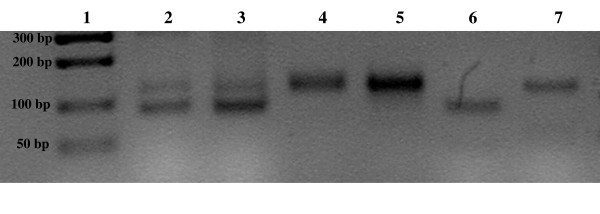
PCR products of bluegill DNA (Lake Wapalanne), employing Lma20 primers to amplify microsatellite regions. Lane 1: molecular weight markers (Hi-Lo Marker, Minnesota Molecular), size indicated in basepairs. Lanes 2–7: bluegill Lma20 microsatellite polymorphisms. Buccal tissues employed for this experiment were stored overnight in 100% ethanol before DNA extraction after 24 hours. Heterozygotes and homozygotes for the Lma20 marker are clearly delineated in the individual fish. 2% agarose gel stained with ethidium bromide. The image was inverted to a negative by Scion computer software (Scion, Inc., Frederick, Maryland).

The quality of the isolated bluegill DNA is high enough to allow PCR amplification of simple sequence repeat polymorphisms without further purification. There is some background visible along with the polymorphic DNA bands, but the bands themselves are clearly visible for each individual fish (Figure 1). Microsatellite regions have been successfully amplified at several bluegill loci (Lma20, Lma21, Lma87, Lma102, Lma120, and Lma124), although data from only the Lma20 locus is presented here (Figure 1). Moreover, we have amplified larger polymorphic microsatellite sequences of 300 basepairs using the marker Lma120 (data not shown). High-resolution agarose gel electrophoretic analysis using comparison to known concentrations of HiLo Marker (Minnesota Molecular, Madison, Minnesota) was employed to determine the size ranges of unamplified genomic DNA. We found molecular weights ranged from 1000 to 7000 basepairs.

**Figure 2:**
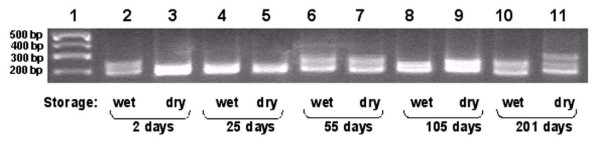
PCR amplification of DNA polymorphisms from aged sunfish tissues, employing Lma20 primers to amplify microsatellite regions. Lane 1: molecular weight markers (Hi-Lo Marker, Minnesota Molecular), size indicated in basepairs. Lanes 2, 4, 6, 8, and 10: PCR amplification of sunfish DNA from tissues that were stored in 100% ethanol. Lanes 3, 5, 7, 9, and 11: PCR amplification of sunfish DNA from tissues that were stored in a dried state. The period of aging is indicated for each set of samples. 2% agarose gel stained with ethidium bromide.

After over 200 days of storage, either preserved in ethanol or in a dried state, the *Lepomis *tissue yielded DNA that amplifies microsatellite sequences quite clearly and strongly (Fig. 2). Additionally, the polymorphisms among the individual bluegills sampled can be identified, suggesting that population studies may be performed even on samples that are months old and stored at room temperature. We conclude that tissue samples taken in this manner are quite stable over long periods either when vacuum dried or left in ethanol.

Note that the PCR products being amplified and examined (Fig. 2) are not from the same individuals, so many of the differences that we are seeing in amplified products arise from polymorphisms between the individuals.

There does appear to be an aberrant product at 201 days between the "wet" and the "dry" preserved samples (Fig. 2, lanes 10 and 11) where an extra band has appeared. There may have been some minor degradation in the dried tissue sample that led to an additional PCR product being generated. It seems more likely that we may be observing two microsatellite (Lma20) alleles, and a heteroduplex band composed of two different size alleles. "Triplet" bands are often produced in PCR, where two basepair repeats are found in the microsatellite sequence, such as in Lma20 (personal communication, Dr. Kirsten Monsen).

The mean concentration of DNA extracted using each method of preservation was comparable: the "wet" preserved DNA was 50.6 ± 7.6 ng/uL, while the "dry" preserved was 46.4 ± 5.1 ng/uL. The range in "dry" concentrations was 29–70 ng/ul. The range of "wet" concentration was 32–69 ng/ul.

It should be noted that although we employed 100% ethanol successfully in our own tissue stability studies, it seems likely that concentrations as low as 50% will work as well [12]. Many researchers may want to consider lowering the ethanol concentration for reasons of economy.

Our findings suggest that valuable molecular genetic data may be obtained not only from piscine tissues that have been stored under optimal conditions, but also from those that have not been stored optimally. Furthermore, it is clear that the lack of adequately low temperatures during transport and long term storage should not be a barrier to anyone wishing to engage in field-based molecular genetic research. Although we have not endeavoured to determine whether tissues gathered in this manner from other aquatic species may have the same longevity of storage, it is likely that tissues obtained from other fish using the described field extraction method will yield robust samples.

## Methods

### Collection and treatment of samples

Bluegill sunfish (*Lepomis macrochirus*) were caught in situ using standard angling equipment and bait of mealworms. The sunfish originated from Lake Wapalanne in northwestern New Jersey. Once caught, the fish were held temporarily (20–90 min) in buckets of lake water.

When ~20 fish were captured, buccal smears were taken from each fish by sterilely swabbing their mouths using the wooden dowel end of sterile cotton-tipped applicators (Moore Medical Corp, New Britain, Connecticut). Cheek cells from the applicator ends were fixed and preserved on site by re-suspension into 100 uL of 100% denatured ethanol (Fisher Scientific, Hanover Park, Illinois) in 1.5 mL microfuge tubes. After taking buccal smears, fish were returned to their lake habitat.

For the study testing the practicality of using buccal swabbing for the tissue collection process, the fixed tissue samples were stored at 4°C for 24–96 hours before extraction.

For the tissue stability study, half the samples were left in ethanol, sealed with parafilm and stored at a constant 26°C (Model E-30B constant temperature chamber, Percival Scientific) in the dark. The remainder of the samples were dried for 20 min on a Savant Speedvac vacuum dryer (GMI, Inc., Albertville, Minnesota) set at the lowest drying temperature. These dried samples were then sealed with parafilm and stored with the ethanol preserved samples in the dark at a constant 26°C. DNA was extracted from the tissues (both ethanol preserved and vacuum dried) after 2, 5, 25, 55, 105, and 201 days.

### DNA extraction and PCR amplification conditions

All ethanol preserved samples were dried for 20 min on a Savant Speedvac vacuum dryer set at the lowest drying temperature. Both "wet" preserved and "dry" preserved tissues were then resuspended in 50 uL of TE and RNase (10 mM Tris-Cl, 1 mM EDTA, pH 8.0, 1 unit RNase per 50 uL aliquot). Tissues were lysed by 5 min incubation at 95°C, cooled on ice for an additional 5 min incubation, and centrifuged briefly to collect condensation.

The polymerase chain reaction was performed employing *Lepomis *amplification primers for detecting microsatellite polymorphisms [14]. The amplification conditions principally followed the directions of Vander Zwan et al [15]. Microsatellites were amplified in 20 uL reactions containing: 29–70 ng fish DNA, 10% ThermoPol buffer (New England Biolabs Inc., Beverly, Massachusetts), 5 pmoles of each primer, 200 uM dNTPs (New England Biolabs Inc., Beverly, Massachusetts), and 1.0 unit Taq polymerase. The primers used for the test were for the Lma20 locus (Lma20F: 5'GGCACTAATCTAATTGTAGCC 3', Lma20R: 5'TTGTGTGTCTGCATTGGAATC 3') [14]. DNA concentrations were determined employing a Nanodrop Spectrophotometer Model ND-1000 (Nanodrop Technologies Inc., Wilmington, Delaware).

All amplification was performed in a Mastercycler Gradient Thermocycler (Eppendorf Inc., Germany). The PCR products were subjected to electrophoresis on a 2% agarose gel in 1X sodium borate buffer [16]. The products on agarose gels were stained with ethidium bromide and imaged using an Ultralum gel documentation system (Ultralum, Inc., Claremont, California) and Scion computer software (Scion, Inc., Frederick, Maryland).

## Authors' contributions

Both JC and JS collected tissue samples, contributed to the experimentation process, and wrote the submitted manuscript.
